# Outcomes of Modified Sistrunk Procedure for Pediatric Thyroglossal Duct Cyst Removal: Systematic Review and Meta‐Analysis

**DOI:** 10.1002/oto2.70308

**Published:** 2026-10-06

**Authors:** Hashim Shaaban, Alma Sato, Annah Waqasi, Carl‐Gabriel McGovern, Ismail Ozgenc, Laith Bayan, James W. F. Burns, Sum‐Yu C. Lee, Christian A. Than, Hayato Nakanishi, Premjit Randhawa

**Affiliations:** ^1^ University College London Hospitals NHS Foundation Trust London UK; ^2^ University of Nicosia Medical School University of Nicosia Nicosia Cyprus; ^3^ Department of Otorhinolaryngology Manchester University NHS Foundation Trust Manchester UK; ^4^ Lewisham and Greenwich NHS trust Queen Elizabeth Hospital London UK; ^5^ Surgical Emergency Unit Oxford University Hospitals NHS Foundation Trust UK; ^6^ The Queen Elizabeth Hospital King's Lynn NHS Foundation Trust Norfolk UK; ^7^ Bolton NHS Foundation Trust Bolton UK; ^8^ University Hospitals of Leicester NHS Trust, Leicester Royal Infirmary Infirmary Square Leicester UK; ^9^ School of Biomedical Sciences The University of Queensland, St Lucia Brisbane Australia; ^10^ Cambridge University Hospitals NHS Foundation Trust Cambridgeshire UK; ^11^ Department of Surgery Mayo Clinic Rochester Minnesota USA; ^12^ Royal National ENT and Eastman Dental Hospitals University College Hospital Grafton Way Building London UK

**Keywords:** complications, modified Sistrunk procedure, pediatric, recurrence, thyroglossal duct cyst

## Abstract

**Objective:**

This meta‐analysis aims to evaluate the safety and efficacy of the modified Sistrunk procedure in pediatric patients with thyroglossal duct cyst.

**Data Sources:**

A comprehensive literature search was conducted across Ovid MEDLINE, Embase, CINAHL and The Cochrane Library, from inception to April 16, 2025.

**Review Methods:**

Screening and selection followed PRISMA guidelines, and the review protocol was prospectively registered with PROSPERO (CRD420251022683). Eligible studies included pediatric patients undergoing the modified Sistrunk procedure for thyroglossal duct cysts. Data extraction was performed independently by reviewers, and study quality was assessed using standardized appraisal tools. Pooled estimates for recurrence, complications, infection, postoperative drainage and operative time were calculated using a random‐effects meta‐analysis model to account for between‐study variability.

**Results:**

Of 999 studies screened, 5 studies met the eligibility criteria, with a total of 937 pediatric patients with thyroglossal duct cyst undergoing modified Sistrunk procedure. The pooled recurrence rate was 4% (95% CI: 0.02, 0.07; *I* ^2^ = 59%), and the complication rate was 12% (95% CI: 0.01, 0.32; *I* ^2^ = 97%). The pooled infection rate was 6% (95% CI: 0.02, 0.10; *I* ^2^ = 84%), whereas the postoperative drainage rate was 5% (95% CI: 0.00, 0.14; *I* ^2^ = 91%), and operative time was 49.0 minutes (95% CI: 33.6 min, 64.5 min; *I* ^2^ = 99%).

**Conclusions:**

The modified Sistrunk procedure appears to be a safe approach for the management of thyroglossal duct cysts for pediatric patients. However, heterogeneity in technique highlights the need for standardized definitions and high‐quality studies to inform clinical guideline development.

Thyroglossal duct cysts (TGDC) are the most common congenital neck lesions, accounting for up to 75% of midline neck masses in children.^1^, ^2^ While some lesions remain clinically quiescent, TGDCs can be complicated by recurrent infection, abscess formation, fistulisation^3^ and malignant transformation.^4^ These complications could increase operative complexity and postoperative morbidity if definitive management is delayed or incomplete. Surgical excision is therefore considered the definitive treatment,^5^ with long‐term success primarily determined by recurrence prevention and complication avoidance.

Sistrunk et al first described his operation in 1920 and published a modified procedure (mSis) in 1928.^6^, ^7^, ^8^ The Sistrunk procedure, which involves cyst excision along with removal of the central hyoid bone and tract up to the foramen cecum, has long been considered the gold standard.^9^ In an effort to reduce morbidity and improve surgical outcomes, various modifications to the Sistrunk technique have been proposed, collectively termed the mSis procedure.^10^, ^11^ These modifications often aim to preserve tongue base musculature or alter the extent of suprahyoid dissection; however, there is no consensus on a surgical approach or guideline for the management of TGDC.^6^, ^12^ Moreover, recurrence rate is influenced by factors such as preoperative infection, cyst number, lesion complexity, and operative technique.^12^, ^13^, ^14^, ^15^ As a result, it is crucial that baseline and perioperative outcomes are reported in a standardized manner to evaluate the safety and efficacy of the modified technique.

Despite the widespread use of mSis procedures, considerable variability exists in how these techniques are defined and reported across studies. To our knowledge, no existing meta‐analysis has evaluated outcomes following the mSis procedure for the management of TGDC. Accordingly, this systematic review and meta‐analysis aimed to synthesize reported clinical outcomes and describe variability in procedural definitions within the existing heterogeneous literature.

## Methods

### Data Search Strategy and Resources

A comprehensive search of several electronic databases from each database's inception to April 16, 2025 was conducted. The databases included Ovid MEDLINE, Embase, CINAHL and The Cochrane Library. An experienced librarian developed and implemented the search strategy with input from the study's principal investigator. Controlled vocabulary supplemented with keywords focused on pediatric patients undergoing mSis procedure for TGDC. The actual strategy listing all search terms used and how they are combined is available in Supplemental Item 1, available online. This study was conducted in compliance with the Preferred Reporting Items for Systematic Reviews and Meta‐analyses (PRISMA) guidelines^16^ and was prospectively registered with PROSPERO (CRD420251022683).

### Eligibility Criteria and Quality Assessment

Eligible studies were studies that met all of the following inclusion criteria: (1) randomized controlled trials (RCTs) or cohort studies; (2) pediatric patients aged <18 years diagnosed with TGDC undergoing the mSis method; and (3) reported on a primary outcome. Case reports, case series, abstracts, conference abstracts, reviews, and articles that were not reported in English were excluded from the study. Additionally, mSis that were not aligned with our definition were excluded.

Article screening and data extraction were conducted by five independent assessors (A.S., A.W., I.O., C.M., J.B.). Each article required agreement from at least two different assessors to be included. The quality of each study was independently evaluated by two authors (A.S., L.B.) using the Risk of bias in non‐randomized studies of interventions (ROBINS‐I).^17^ Any disagreements in screening and quality assessments were adjudicated by a third author (H.S.) and discussed with co‐authors as necessary.

### Extracted Primary Outcomes and Definitions

Primary outcomes included recurrence rates and overall complications. Secondary outcomes consisted of operative time, follow‐up duration, postoperative complications, perioperative infection rate, and postoperative drainage rate. In our meta‐analysis mSis procedure was defined as a modification of the standard technique that includes sparing of the tongue root/base/cecum,^10^, ^11^, ^18^, ^19^, ^20^ which represented the most commonly reported procedural variation. This definition was applied to maximize methodological consistency within the pooled data set. Different ‘modified’ approaches were extracted and presented in tabulated form (Table 1) to summarize the slight procedural variations reported across studies. Other procedural variations, including extended resections, endoscopic approaches, and alternative minor modifications, were each reported in only one or two studies, and therefore, subgroup analysis based on specific procedural variations was not feasible.

**Table 1:** Summary of Different Definitions of Modified Sistrunk Procedure

Author, Year	Name of method	Description
Arda 2021, Brooks 2019, Gumussoy 2021, Li 2024, Pardi 2020, Pucher 2018, Pupic‐Bakrac 2023, Wang 2023	“Tongue base sparing”	This procedure involves coring out the hyoid bone and the fistula beyond. However, the tongue root (foramen cecum) is spared. This description may sometimes be used interchangeably with “muscle sparing.”
Ahmed 2011	“Extended”	The classical dissection is extended laterally from the main duct territory and includes a block of tissue incorporating the infrahyoid section. (May include tongue base breach).
Anuwong 2017	“Endoscopic/minimally invasive”	This approach involves an incision made in the axilla and breast to remove the cyst, the central hyoid bone, and the tract via endoscopic techniques
Hussain 2013, Maddalozzo 2010, Ryu 2015, Zhang 2011	“Other”	Different modifications were noted in literature; however, foramen cecum was cored out. Ryu et al, describes the use of monopolar Bovie electro‐cauterization for hyoid bone dissection. Zhang et al, mentions the use of methylene blue dye for tracing of cysts. Maddalozzo et al, describes further coring out of the posterior hyoid space.

### Statistical Analysis

Proportions of data were pooled using the generic inverse variance method of DerSimonian and Laird. Proportions underwent logit transformation prior to meta‐analysis. Between‐study variance *τ* ^2^ was calculated using the restricted maximum likelihood (REML) method. The heterogeneity of effect size estimates across the studies was quantified using the *Q* statistic and *I* ^2^. A value of *I* ^2^ of 0% to 25% indicates insignificant statistical heterogeneity, 26% to 50% low heterogeneity, and 51% to 100% high heterogeneity.^21^ When mean and standard deviation (SD) were unavailable, the median was converted to mean using the formulas from the Cochrane Handbook for Systematic Reviews of Interventions.^22^ If the SD was not available or extractable, the reported mean was omitted from the calculation. Data analysis and forest plot generation was performed in the RStudio® 2024.04.2 environment for R 4.4.0 using the “meta” package.

## Results

### Study Selection and Patient Characteristics

The initial literature search of the electronic databases yielded 999 studies. After removing duplications and applying the eligibility criteria, 59 studies were retained for full‐text review. Five unique studies involving 937 pediatric patients who fit our inclusion criteria were included in this meta‐analysis (Supplemental Item 2, available online). These studies were all retrospective, of which three were multicenter studies^10^, ^11^, ^19^ and two were single‐center studies.^18^, ^20^ The mean age of participants across studies ranged from 2.5 to 5.4 years, and 405 (43%) patients were female. The baseline characteristics of the included studies are described in Table 2.

**Table 2:** Baseline Characteristics of Included Studies

First author, year	Country	Study setting	Sample Size (n)	Female: n (%)		Average age at surgery ± SD [years]
				Total	Included mSis	
Arda, 2021	US/ Turkey	MC	105	84 (53.5)	58 (55)	5.3 ± 4.5
Li, 2024	China	MC	391	147 (37.6)	147 (37.6)	5.4 ± 2.8
Pardi, 2020	Italy	SC	57	23 (40)	23 (40)	5.0 ± 2.5
Pupic‐Bakrac, 2023	Croatia/Bosnia‐Herzegovina	MC	44	48 (43.6)	NR	NR
Wang, 2023	China	SC	340	153 (45)	153 (45)	3.7 ± 2.3

Abbreviations: MC, multicentre; mSis, modified Sistrunk; NR, not reported; SC, single center.

### Risk of Bias

Of the cohort studies judged via ROBINS‐I tool, three were classified as moderate risk,^10^, ^11^, ^20^ and two as serious risk.^18^, ^19^ Serious risk was primarily attributed to confounding missing data, and measurement of outcomes^18^, ^19^ due to non‐randomized allocation and inadequate adjustment for key confounders such as baseline disease severity and prior treatments. Nonetheless, all studies were deemed eligible for analysis as patients appeared to represent the whole experience of the investigator. The exposure and outcomes were adequately ascertained, and the lengths of follow‐up were adequate for the purposes of this study. Results of the quality assessment of all included studies are shown in Supplemental Item 3, available online.

### Clinical characteristics

Among the five included studies, a total of 937 pediatric patients underwent an mSis procedure. Three studies^10^, ^11^, ^20^ included the type of TGDC, demonstrating 323 patients (83%) with a single cyst,^11^ 68 patients (17%) with multiple,^11^ and 73 (9%) with prior incision and drainage. Mean follow‐up duration ranged from 6 to 84 months. The clinical characteristics of the included studies are summarized in Table 3.

**Table 3:** Clinical Characteristics of the Included Studies

First author, year	Country	Singlecentre [SC] /multicentre [MC]	Number of cysts (n)	Prior incision and drainage (n)	Mean length of hospital stay (days)	Mean follow‐up duration (months)
Arda, 2021	US/ Turkey	MC	Single cyst: 7(R) 145 (no R) Multiple cysts 1(R) 4 (no R)	15	NR	54.5
Li, 2024	China	MC	Single cyst: 323 Multiple Cysts: 68	40	14.3	69.6
Pardi, 2020	Italy	SC	NR	NR	NR	55
Pupic‐Bakrac, 2023	Croatia/Bosnia‐Herzegovina	MC	NR	NR	NR	84
Wang, 2023	China	SC	NR	18	NR	6

Abbreviations: MC, multi‐center; n, number of cases; R, recurrence; SC, single center.

### Outcomes

The recurrence rate from five studies^10^, ^11^, ^18^, ^19^, ^20^ was 4% (95% CI: 0.02, 0.07; *I* ^2^ = 59%, n = 937). The overall complication rate across four studies^10^, ^11^, ^18^, ^20^ was 12% (95% CI: 0.01, 0.32; *I* ^2^ = 97%, n = 893). The surgical site infection rate from three studies^10^, ^11^, ^20^ was 6% (95% CI: 0.02, 0.10; *I* ^2^ = 84%, n = 836). Furthermore, postoperative drainage rate from three studies^10^, ^11^, ^18^ was 5% (95% CI: 0.00, 0.14; *I* ^2^ = 91%, n = 553). The operative time from two studies^18^, ^20^ was 49.0 minutes (95% CI: 33.6, 64.5; *I* ^2^ = 99%, n = 397). The outcomes are summarized in 1, 2, 3, 4, 5.

**Figure 1:**
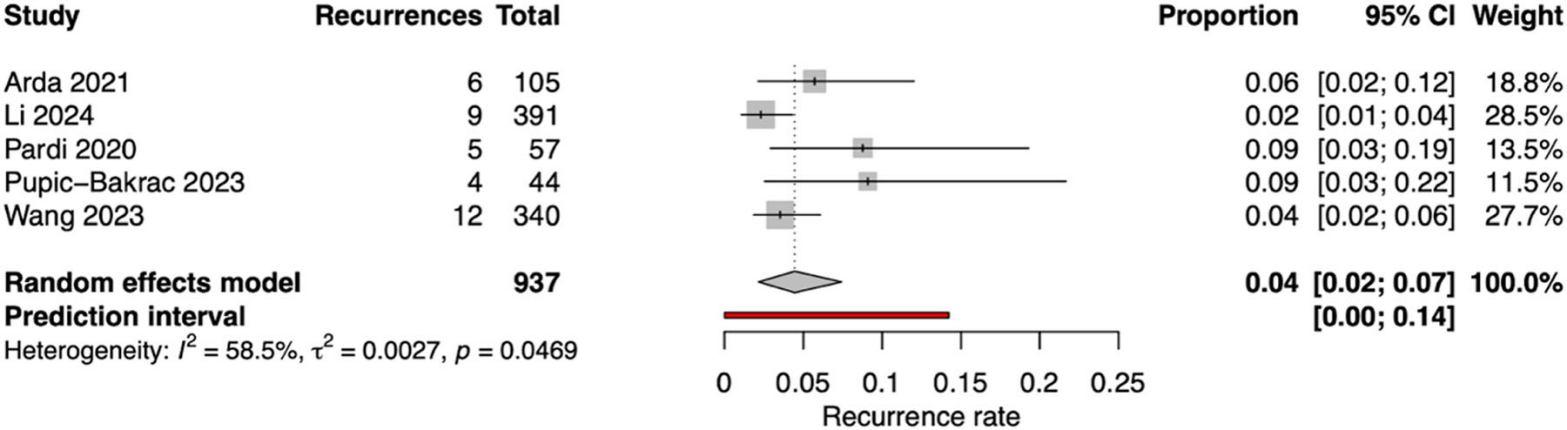
Pooled estimate of recurrence rates.

**Figure 2:**
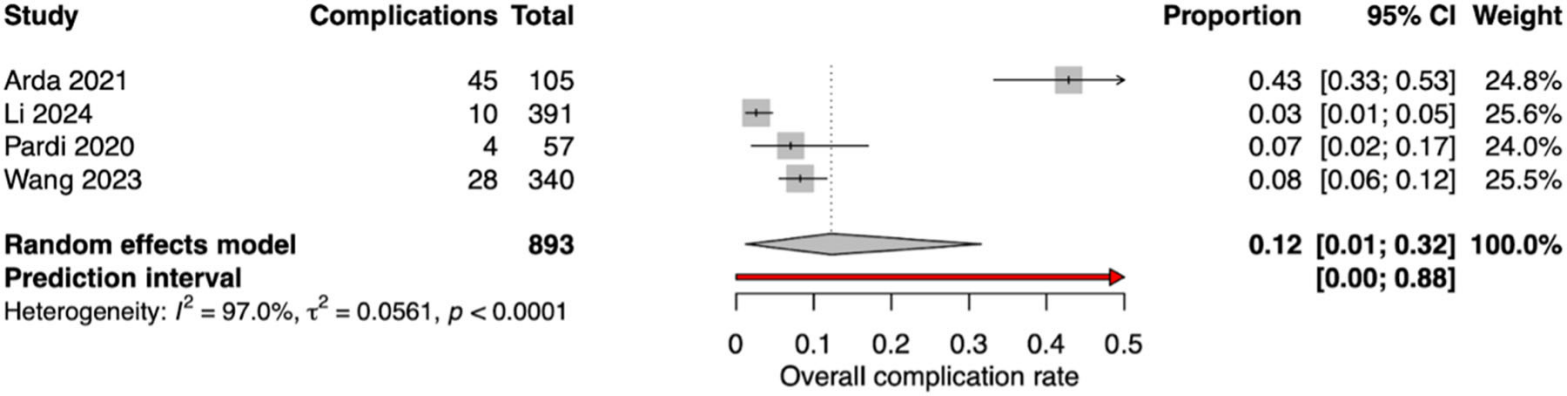
Pooled estimate of overall complications.

**Figure 3:**
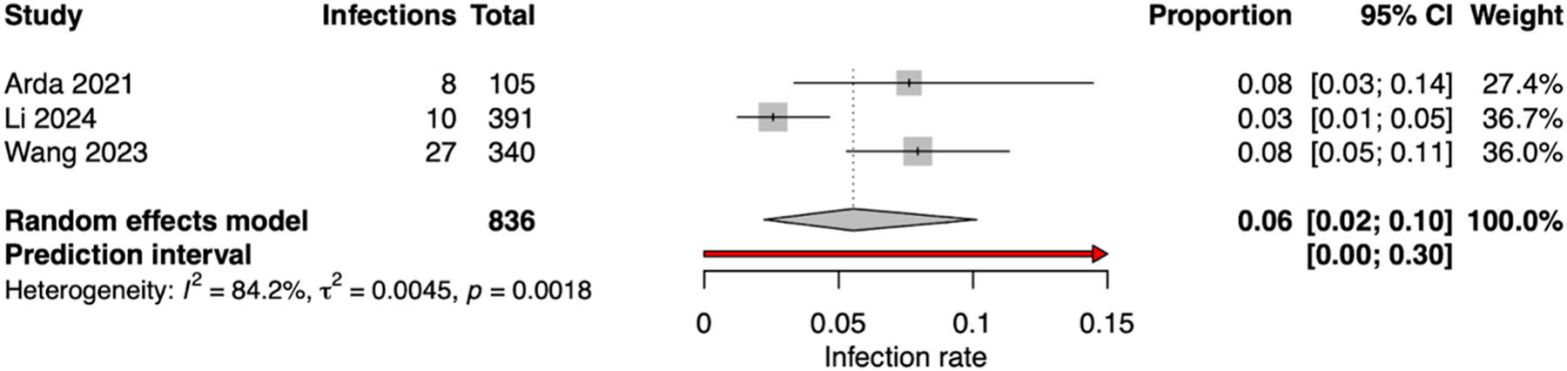
Pooled estimate of surgical site infection rates.

**Figure 4:**
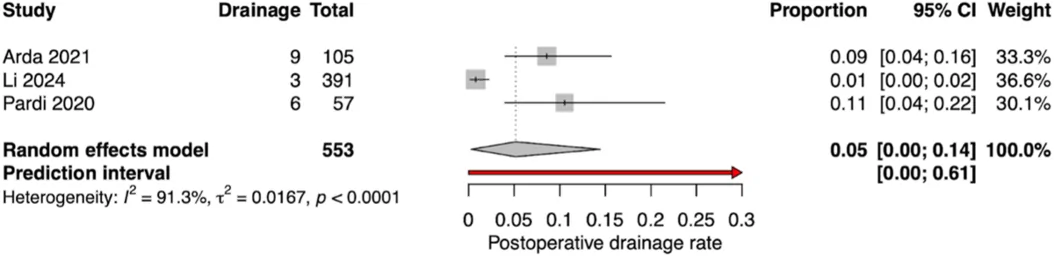
Pooled estimate of postoperative drainage.

**Figure 5:**
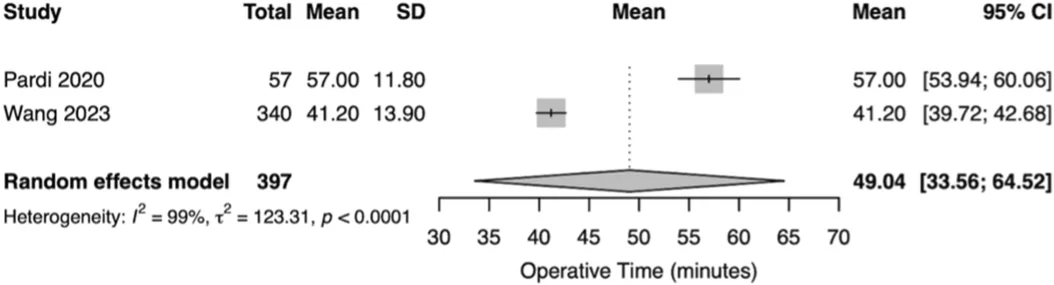
Pooled estimate of operative time.

## Discussion

The mSis procedure has gained increasing attention as a surgical refinement aimed at improving safety and efficacy in the excision of TGDC in pediatric population. Although the use of muscle‐sparing mSis approach increased, uncertainty remains regarding its recurrence risk and the influence of factors such as preoperative infection and anatomical variability. In this meta‐analysis, pooled estimates suggest low reported recurrence and complication rates. However, variability in procedural definitions and inconsistent reporting of clinical variables limited more detailed interpretation of predictors of recurrence, highlighting the need for standardized definitions and reporting in future studies.

A central rationale of the mSis procedure is that targeted excision of the thyroglossal duct tract, while preserving surrounding musculature, may reduce the presence of residual epithelial remnants without increasing recurrence risk. In our meta‐analysis, the pooled recurrence rate was 4%, although recurrence rates varied across the existing literature describing mSis techniques.^10^, ^23^, ^24^, ^25^, ^26^ These variations likely reflect differences in surgeons’ techniques aimed at balancing complete duct excision with preservation of surrounding structures. For example, some authors emphasize extending the resection superiorly into the suprahyoid space to address potential residual tracts,^27^, ^28^ whereas others describe excision margins guided by anatomical landmarks such as the pre‐tracheal^29^ or more extensive clearance of the posterior hyoid space.^30^ While recurrence outcomes appeared low within our tongue base sparing subset, other procedural modifications were reported in only one or two studies, precluding subgroup analysis and limiting definitive conclusions regarding recurrence across all modified techniques. Accordingly, these findings should be interpreted with caution. Currently, no guideline clearly defines the mSis procedure or its anatomical boundaries, which may partly explain differences in reported recurrence outcomes and highlights the need for standardized definitions of the procedure.^30^, ^31^

These variations are summarized in Table 1, illustrating the diversity of modifications reported in the literature. For the purpose of analysis, a muscle‐sparing modification preserving the tongue root or base was used as the operational definition to enable pooling of available data. While recurrence outcomes were low within this subset, the limited number of eligible studies underscores the need for standardized procedural definitions to enable more robust comparisons and clearer interpretation of recurrence risk.

The role of preoperative infection in TGDC has long been debated, with some studies suggesting it may increase the risk of recurrence. Although all included studies reported preoperative infection status, affecting 292 patients (29%), recurrence outcomes could not be extracted stratified by infection status. Among the included studies, the majority reported no clear association between preoperative infection and postoperative TGDC recurrence,^10^, ^19^, ^20^ while one study suggested a higher recurrence risk in infected cases.^11^ In contrast, several existing studies have reported an association between preoperative infection and increased recurrence risk.^31^, ^32^, ^33^ This association may be explained by inflammatory changes during active infection, as tissue edema and congestion can obscure lesion boundaries and hinder complete excision while leaving a thyroglossal duct remnant. Moreover, inflammation also increases the risk of fistula wall or branch rupture during dissection, both of which may contribute to recurrence.^11^ Additionally, inflammatory changes may persist after clinical resolution of infection, and recent preoperative cyst infection may still influence surgical outcomes through residual inflammation at the time of excision.^31^ Antibiotics administered during the acute inflammatory phase, when patients present to clinics or preoperatively, have been suggested to reduce postoperative recurrence.^26^, ^34^ Within the context of the muscle‐sparing mSis technique evaluated in this meta‐analysis, no clear association between preoperative infection and recurrence was observed in the available data.^10^, ^19^, ^20^ Collectively, these findings highlight the need for standardized reporting of infection timing, operative findings, and recurrence outcomes to clarify whether preoperative infection represents an independent risk factor.

Another factor that could contribute to increased recurrence risk is the number of cysts. In our study, only two studies explicitly addressed these features.^10^, ^11^ Within this context, cyst number has been inconsistently reported across studies evaluating mSis procedures, and interpretation of recurrence patterns in relation to cyst number therefore remains limited.^35^, ^36^, ^37^ This limitation is particularly pertinent, as the intrinsic anatomical complexity of the thyroglossal duct system may influence both recurrence risk and apparent adequacy of surgical excision. Existing imaging, histological, and cadaveric studies consistently demonstrate substantial variability in thyroglossal duct morphology, including accessory tracts, arborising ductules, and atypical tract courses that may not be readily appreciable intraoperatively, even when standard anatomical landmarks are respected.^27^, ^34^, ^38^, ^39^, ^40^ Marianowski et al^13^ reported that preoperative cystic formations may influence recurrence risk, with recurrence rates of 3.8% for single cysts and 47% for multiple cysts following the traditional Sistrunk procedure. This observation underscores the potential clinical relevance of cyst number and highlights the need for consistent reporting of this variable in future studies. Taken together, these findings suggest that recurrence outcomes following mSis should be interpreted in the context of anatomical variability and reporting limitations rather than as evidence of procedural superiority.

### Limitations

It is imperative to acknowledge the limitations of our study. First, all included studies were retrospective cohort studies, with no RCTs available for comparison. Second, there was a lack of standardized reporting of baseline and perioperative characteristics such as operative time, cyst number, length of hospital stay, and estimated blood loss among the included studies. Third, heterogeneity in the definition of the “modified” Sistrunk technique limited inclusion to this meta‐analysis. The inclusion criteria required explicit description in the mSis procedure that describes sparing of the tongue base or musculature, as this represented the most commonly reported modification and provided sufficient data for quantitative synthesis. Other modified techniques were identified but were reported too infrequently to permit meaningful pooled analysis. This could have reduced the sample size and limited the generalizability of the findings to all mSis procedures. Furthermore, the absence of sufficient comparative studies between traditional and mSis procedures precluded comparative meta‐analysis and limits conclusions regarding relative effectiveness. Additionally, follow‐up duration varied substantially between studies, potentially leading to underestimation of true recurrence rates and delayed postoperative complications. Finally, important confounding factors, such as preoperative infection status, drain use, surgeon's expertise, perioperative antibiotic protocols and cyst number, were inconsistently reported, limiting the ability to perform subgroup or sensitivity analyses. Future prospective studies with a standardized definition and long‐term follow‐up are needed to address these limitations and better characterize outcomes following the mSis procedure.

## Conclusion

This meta‐analysis provides a synthesis of the available literature describing outcomes following the mSis procedure in pediatric patients with TGDC. While pooled estimates suggested relatively low reported recurrence and complication rates within the included studies, the limited number of studies, heterogeneity in procedural definitions, and inconsistent reporting of baseline and perioperative variables preclude definitive conclusions regarding the efficacy of the technique. Furthermore, the lack of sufficient comparative data does not permit conclusions regarding the relative performance of mSis compared with the traditional Sistrunk procedure. These findings highlight the need for standardized definitions, comprehensive baseline reporting, and prospective comparative studies to better characterize outcomes following mSis and inform future clinical practice.

## Author Contributions

**Hashim Shaaban,** conceived and designed the study, developed the study protocol, adjudicated disagreements during screening and quality assessment, performed data analysis and interpretation, and drafted the manuscript; **Alma Sato,** conducted article screening, data extraction, and data analysis; performed quality assessment; and contributed to manuscript revision; **Annah Waqasi,** conducted article screening and data extraction and contributed to manuscript drafting; **Ismail Ozgenc,** conducted article screening and data extraction and contributed to manuscript drafting; **Carl‐Gabriel McGovern,** conducted article screening and data extraction and contributed to manuscript revision; **James W. F. Burns,** conducted article screening and data extraction and contributed to manuscript revision; **Laith Bayan,** performed quality assessment and contributed to data analysis, data interpretation, and manuscript drafting; **Sum‐Yu C. Lee,** conducted article screening and contributed to manuscript revision; **Christian A. Than,** conceived and designed the study, developed the study protocol, supervised the study, and critically revised the manuscript for important intellectual content; **Hayato Nakanishi,** conceived and designed the study, developed the study protocol, and contributed to data interpretation and manuscript revision; **Premjit Randhawa,** conceived and designed the study, developed the study protocol, provided senior oversight, and critically revised the manuscript; all authors have approved the final version of the manuscript and agree to be accountable for all aspects of the work.

## Disclosures

### Competing interests

All authors declare that they have no conflicts of interest relevant to the subject matter discussed in this manuscript. No author has any financial or personal relationships with organizations that could inappropriately influence or bias the work presented. Specifically, none of the authors are employed by, holds stock in, serves on advisory boards.

### Funding source

None.

## Supporting information


**Supplemental Item 1:** Search Strategy.

**Supplemental Item 2:** PRISMA flowchart of the study selection process.

**Supplemental Item 3:** Risk of bias in non‐randomized studies of interventions (ROBINS‐I) assessment.
