# Supplementary material for: Outcomes of Modified Sistrunk Procedure for Pediatric Thyroglossal Duct Cyst Removal: Systematic Review and Meta‐Analysis
Source: OTO Open. 2026 Oct 6;10(4):e70308. doi: 10.1002/oto2.70308 (PMC13640523; doi:10.1002/oto2.70308)
Supplement: Supplementary file 1 [file OTO2-10-e70308-s003.pdf]

PRISMA 2020 flow diagram for new systematic reviews which included searches of databases, registers and other sources

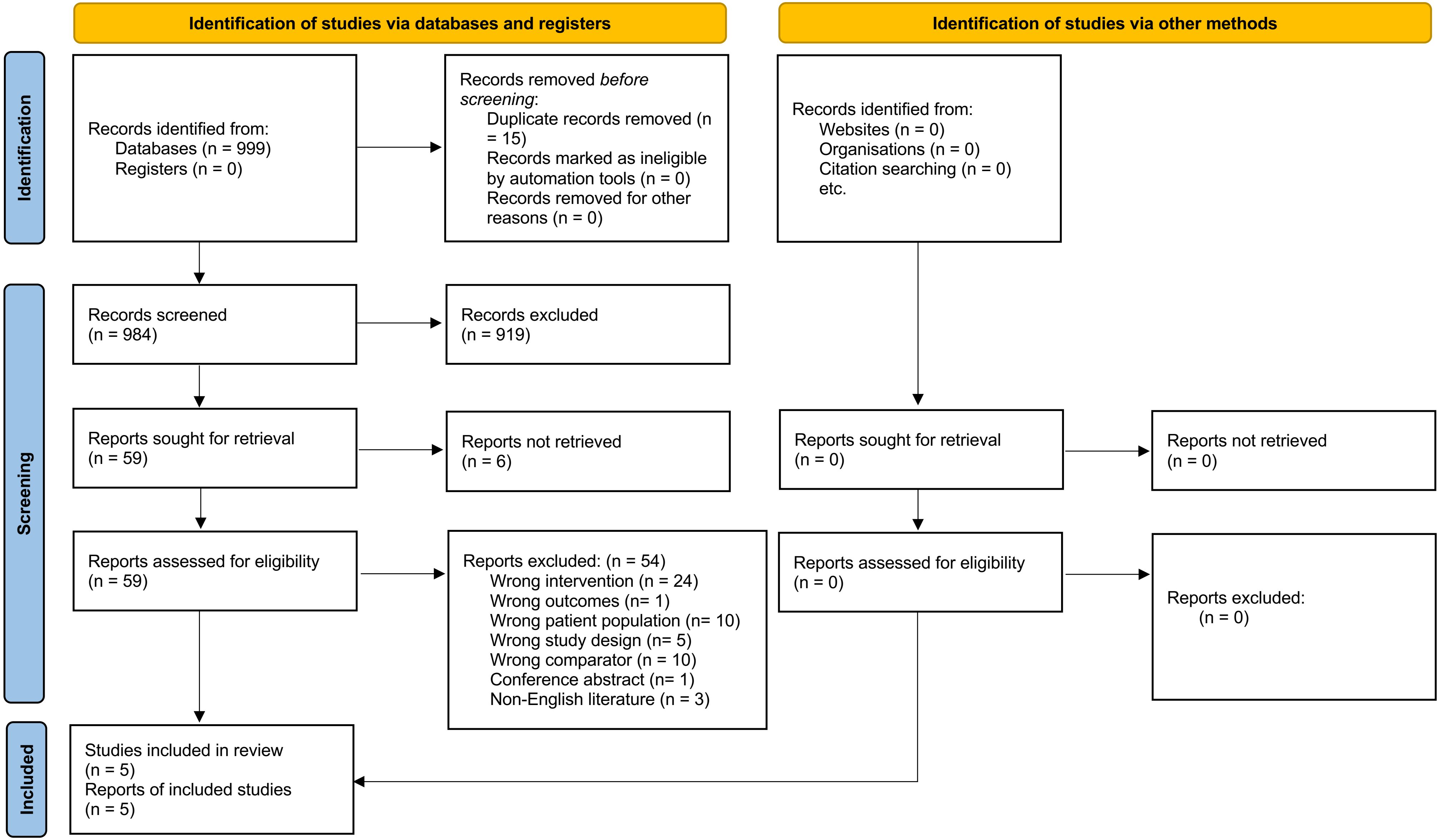

*Consider, if feasible to do so, reporting the number of records identified from each database or register searched (rather than the total number across all databases/registers).
**If automation tools were used, indicate how many records were excluded by a human and how many were excluded by automation tools.

From: Page MJ, McKenzie JE, Bossuyt PM, Boutron I, Hoffmann TC, Mulrow CD, et al. The PRISMA 2020 statement: an updated guideline for reporting systematic reviews. BMJ 2021;372:n71. doi: 10.1136/bmj.n71.
