# Supplementary material for: Outcomes of Modified Sistrunk Procedure for Pediatric Thyroglossal Duct Cyst Removal: Systematic Review and Meta‐Analysis
Source: OTO Open. 2026 Oct 6;10(4):e70308. doi: 10.1002/oto2.70308 (PMC13640523; doi:10.1002/oto2.70308)
Supplement: Supplementary file 2 [file OTO2-10-e70308-s002.docx]

**Supplemental Item 1: Search Strategy**

**Search strategies**: Thyroglossal Duct Cyst. Databases used: Medline- (449 results), Embase – (559 results), CINAHL- (102 results), The Cochrane Library (114 results; 7 reviews, 107 trials). Total number of results 1224. Duplicates removed – 225. Total remaining – 995.

**Medline**

**Ovid MEDLINE(R) ALL** <1946 to April 16, 2025>

1          	(Modified Sistrunk Procedure* or mSis).ti,ab.   924

2          	surgical procedure*.ti,ab.        	122956

3          	Surgical Procedures, Operative/          	57531

4          	neck dissection.ti,ab.	11145

5          	Neck Dissection/         	8912

6          	otorhinolaryngological surgical procedure*.ti,ab.       	7

7          	Otorhinolaryngologic Surgical Procedures/   	5511

8          	(Thyroglossal Duct Cyst* or TGDC).ti,ab.         	1049

9          	Thyroglossal Cyst/      	1422

10       	Thyroglossal Cyst*.ti,ab.           	334

11       	thyroid cartilage.ti,ab.   1734

12       	thyroid gland*.ti,ab.   	27330

13       	Thyroid Cartilage/       	1467

14       	Thyroid Gland/  61410

15       	thyroid bone.ti,ab.      	53

16       	8 or 9 or 10 or 11 or 12 or 13 or 14 or 15            	80588

17       	drainage.ti,ab.   115613

18       	Drainage/        	48560

19       	1 or 2 or 3 or 4 or 5 or 6 or 7 or 18         	244571

20       	risk factor*.ti,ab.          	856737

21       	Risk Factors/	1028420

22       	treatment outcome*.ti,ab.      	78822

23       	Treatment Outcome/	1243110

24       	ossification.ti,ab.        	23662

25       	Osteogenesis/  47741

26       	reoperation.ti,ab.        	39894

27       	Reoperation/	99503

28       	Hemorrhage/	86280

29       	bleeding.ti,ab.  265650

30       	cyst* infect*.ti,ab.       	683

31       	Neoplasm Recurrence, Local/  153787

32       	Neoplasm Recurrence.ti,ab. 	78

33       	20 or 21 or 22 or 23 or 24 or 25 or 26 or 27 or 28 or 29 or 30 or 31 or 32             	3143202

34       	16 and 19 and 33         	410

35       	Sistrunk Procedure*.ti,ab.       	250

36       	19 or 35            	244742

37       	16 and 33 and 36         	441

38       	"Length of Stay"/          	110098

39       	length of stay.ti,ab.     	89928

40       	operation duration.ti,ab.          	1690

41       	33 or 38 or 39 or 40    	3242237

42       	16 and 36 and 41         	449

**Embase**

Embase <1974 to 2025 April 16>

1          	(Modified Sistrunk Procedure* or mSis).ti,ab.   1471

2          	surgical procedure*.ti,ab.        	168439

3          	surgical technique/    	421906

4          	neck dissection.ti,ab.	15042

5          	neck dissection/          	21228

6          	otorhinolaryngological surgical procedure*.ti,ab.       	7

7          	ear nose throat surgery/           	4915

8          	drainage.ti,ab.   162717

9          	wound drainage/ or surgical drainage/ 35139

10       	1 or 2 or 3 or 4 or 5 or 6 or 7 or 8 or 9  	723221

11       	(Thyroglossal Duct Cyst* or TGDC).ti,ab.         	1318

12       	thyroglossal duct cyst/ 2108

13       	Thyroglossal Cyst*.ti,ab.           	386

14       	thyroid cartilage.ti,ab.   2219

15       	thyroid cartilage/         	1522

16       	thyroid gland*.ti,ab.   	30092

17       	thyroid bone.ti,ab.      	95

18       	11 or 12 or 13 or 14 or 15 or 16 or 17 	34975

19       	risk factor*.ti,ab.          	1260139

20       	risk factor/      	1511310

21       	treatment outcome*.ti,ab.      	116870

22       	treatment outcome/ 	1034308

23       	ossification.ti,ab.        	28237

24       	ossification/  	58764

25       	reoperation.ti,ab.        	55032

26       	reoperation/  	115936

27       	bleeding.ti,ab.  436282

28       	bleeding/         	380134

29       	cyst* infect*.ti,ab.       	973

30       	tumor recurrence/      	75063

31       	Neoplasm Recurrence.ti,ab. 	100

32       	19 or 20 or 21 or 22 or 23 or 24 or 25 or 26 or 27 or 28 or 29 or 30 or 31             	3719150

33       	10 and 18 and 32         	479

34       	"length of stay"/            	313318

35       	operation duration/    	169398

36       	length of stay.ti,ab.     	169454

37       	operation duration.ti,ab.          	2370

38       	32 or 34 or 35 or 36 or 37         	4019118

39       	10 and 18 and 38         	559

**CINAHL**

| # | Query | Last Run Via | Results |
| --- | --- | --- | --- |
| S39 | S18 AND S21 AND S38 | Interface - EBSCOhost Research Databases Search Screen - Advanced Search Database - CINAHL | 102 |
| S38 | S22 OR S23 OR S24 OR S25 OR S26 OR S27 OR S28 OR S29 OR S30 OR S31 OR S32 OR S33 OR S34 OR S35 OR S36 OR S37 | Interface - EBSCOhost Research Databases Search Screen - Advanced Search Database - CINAHL | 930,957 |
| S37 | XB operation duration | Interface - EBSCOhost Research Databases Search Screen - Advanced Search Database - CINAHL | 373 |
| S36 | (MH "Length of Stay") | Interface - EBSCOhost Research Databases Search Screen - Advanced Search Database - CINAHL | 53,930 |
| S35 | XB Length of Stay | Interface - EBSCOhost Research Databases Search Screen - Advanced Search Database - CINAHL | 35,266 |
| S34 | XB Neoplasm Recurrence | Interface - EBSCOhost Research Databases Search Screen - Advanced Search Database - CINAHL | 3,424 |
| S33 | (MH "Neoplasm Recurrence, Local") | Interface - EBSCOhost Research Databases Search Screen - Advanced Search Database - CINAHL | 27,250 |
| S32 | XB cyst* infect* | Interface - EBSCOhost Research Databases Search Screen - Advanced Search Database - CINAHL | 88 |
| S31 | (MH "Hemorrhage") | Interface - EBSCOhost Research Databases Search Screen - Advanced Search Database - CINAHL | 19,738 |
| S30 | XB bleeding | Interface - EBSCOhost Research Databases Search Screen - Advanced Search Database - CINAHL | 51,220 |
| S29 | (MH "Reoperation") | Interface - EBSCOhost Research Databases Search Screen - Advanced Search Database - CINAHL | 21,950 |
| S28 | XB reoperation | Interface - EBSCOhost Research Databases Search Screen - Advanced Search Database - CINAHL | 8,135 |
| S27 | (MH "Osteogenesis") | Interface - EBSCOhost Research Databases Search Screen - Advanced Search Database - CINAHL | 4,780 |
| S26 | XB ossification | Interface - EBSCOhost Research Databases Search Screen - Advanced Search Database - CINAHL | 4,121 |
| S25 | (MH "Treatment Outcomes") | Interface - EBSCOhost Research Databases Search Screen - Advanced Search Database - CINAHL | 448,649 |
| S24 | XB treatment outcome* | Interface - EBSCOhost Research Databases Search Screen - Advanced Search Database - CINAHL | 22,713 |
| S23 | (MH "Risk Factors") | Interface - EBSCOhost Research Databases Search Screen - Advanced Search Database - CINAHL | 195,994 |
| S22 | XB risk factor* | Interface - EBSCOhost Research Databases Search Screen - Advanced Search Database - CINAHL | 239,178 |
| S21 | S8 OR S19 OR S20 | Interface - EBSCOhost Research Databases Search Screen - Advanced Search Database - CINAHL | 72,254 |
| S20 | (MH "Drainage") | Interface - EBSCOhost Research Databases Search Screen - Advanced Search Database - CINAHL | 7,918 |
| S19 | XB drainage | Interface - EBSCOhost Research Databases Search Screen - Advanced Search Database - CINAHL | 15,511 |
| S18 | S9 OR S10 OR S11 OR S12 OR S13 OR S14 OR S15 OR S16 OR S17 | Interface - EBSCOhost Research Databases Search Screen - Advanced Search Database - CINAHL | 5,918 |
| S17 | XB thyroid bone | Interface - EBSCOhost Research Databases Search Screen - Advanced Search Database - CINAHL | 4 |
| S16 | (MH "Thyroid Gland") | Interface - EBSCOhost Research Databases Search Screen - Advanced Search Database - CINAHL | 3,943 |
| S15 | XB thyroid gland* | Interface - EBSCOhost Research Databases Search Screen - Advanced Search Database - CINAHL | 1,799 |
| S14 | XB thyroid cartilage | Interface - EBSCOhost Research Databases Search Screen - Advanced Search Database - CINAHL | 368 |
| S13 | (MH "Thyroid Cartilage") | Interface - EBSCOhost Research Databases Search Screen - Advanced Search Database - CINAHL | 454 |
| S12 | XB thyroid cartilage | Interface - EBSCOhost Research Databases Search Screen - Advanced Search Database - CINAHL | 368 |
| S11 | XB Thyroglossal Cyst* | Interface - EBSCOhost Research Databases Search Screen - Advanced Search Database - CINAHL | 28 |
| S10 | (MH "Thyroglossal Cyst") | Interface - EBSCOhost Research Databases Search Screen - Advanced Search Database - CINAHL | 32 |
| S9 | XB (Thyroglossal Duct Cyst* or TGDC) | Interface - EBSCOhost Research Databases Search Screen - Advanced Search Database - CINAHL | 214 |
| S8 | S1 OR S2 OR S3 OR S4 OR S5 OR S6 OR S7 | Interface - EBSCOhost Research Databases Search Screen - Advanced Search Database - CINAHL | 54,090 |
| S7 | (MH "Surgery, Otorhinolaryngologic") | Interface - EBSCOhost Research Databases Search Screen - Advanced Search Database - CINAHL | 3,039 |
| S6 | TI otorhinolaryngological surgical procedure* | Interface - EBSCOhost Research Databases Search Screen - Advanced Search Database - CINAHL | 0 |
| S5 | (MH "Neck Dissection") | Interface - EBSCOhost Research Databases Search Screen - Advanced Search Database - CINAHL | 1,814 |
| S4 | XB neck dissection | Interface - EBSCOhost Research Databases Search Screen - Advanced Search Database - CINAHL | 2,280 |
| S3 | (MH "Surgery, Operative") | Interface - EBSCOhost Research Databases Search Screen - Advanced Search Database - CINAHL | 26,780 |
| S2 | XB surgical procedure* | Interface - EBSCOhost Research Databases Search Screen - Advanced Search Database - CINAHL | 22,545 |
| S1 | XB ((Modified Sistrunk Procedure* or mSis)) | Interface - EBSCOhost Research Databases Search Screen - Advanced Search Database - CINAHL | 307 |
